# 

**Published:** 1989-08

**Authors:** 


Copyright © Clinical Press Ltd 1989.

**Contents**

62 **Editorial**
63 **Richard Bright—a man of many parts**
J. Campbell MacKenzie
67 **Obituary—Dr W. H. Hylton**
68 **An alternative approach to sex offenders**
Hugh W. Gilbert, J. Clive Gingell
70 **Asymptomatic and recurrent hyperparathyroidism**
J. R. Farndon
72 **The Bristol Children's Hospital's experience of Tracho-bronchial Foreign bodies 1977–1987**
A. H. Hamilton, F. Carswell, J. D. Wisheart
75 **GPs and Consultants: is there agreement on patient management?**
Michael J. Whitfield, Morag Bradley
79 **Shakespeare—a new discovery**
Martin Crosfill
80 **Psychopathology in Art—meditation on Francis Bacon**
Michael Wilson
82 **Splendid but hopeless**
J. L. Burton
83 **A study on the level of knowledge of health matters among patients in a rural practice**
A. D. L. Austin
85 **Surgical Club of South West England—Meeting in Barnstaple, Oct. 1988**
87 **South West Orthopoedic Club—Meeting in Truro, April 1988.**

